# Biodegradable Guided Bone Regeneration Membranes for Periodontal Regeneration in Dogs

**DOI:** 10.3390/dj14080522

**Published:** 2026-08-14

**Authors:** Laura Costa Pinho, Catarina Santos, Maria Helena Fernandes, Bruno Colaço

**Affiliations:** 1 BoneLab, Faculdade de Medicina Dentária, Universidade do Porto, Rua Dr. Manuel Pereira da Silva, 4200-393 Porto, Portugal; 2 LAQV—Laboratório Associado para a Química Verde/REQUIMTE, Faculdade de Medicina Dentária, Universidade do Porto, Rua Dr. Manuel Pereira da Silva, 4200-393 Porto, Portugal; 3 CITAB—Centre for the Research and Technology of Agro-Environmental and Biological Sciences, Inov4Agro, Universidade de Trás-os-Montes e Alto Douro, Quinta de Prados, 5001-801 Vila Real, Portugal; 4 CQE—Centro de Química Estrutural, Institute of Molecular Sciences (IMS), Instituto Superior Técnico, Universidade de Lisboa, Av. Rovisco Pais 1, 1049-001 Lisbon, Portugal; 5 Escola Superior de Tecnologia de Setúbal, Instituto Politécnico de Setúbal, Campus IPS—Estefanilha, 2910-761 Setúbal, Portugal; 6 CECAV—Animal and Veterinary Research Centre, Universidade de Trás-os-Montes e Alto Douro, Quinta de Prados, 5001-801 Vila Real, Portugal; 7 Associate Laboratory for Animal and Veterinary Sciences (AL4AnimalS), Universidade de Trás-os-Montes e Alto Douro, Quinta de Prados, 5001-801 Vila Real, Portugal

**Keywords:** guided bone regeneration, biodegradable membranes, regenerative therapy, veterinary dental health, canine periodontal disease

## Abstract

Guided bone regeneration (GBR) is a widely used clinical approach for managing bone defects associated with periodontal disease in dogs, employing barrier membranes to selectively direct tissue regeneration. The performance of these membranes is influenced by composition, structural design, and degradation behavior, which together determine biological responses and clinical outcomes. Biodegradable GBR membranes, fabricated from natural polymers, synthetic polymers, or composite materials, offer advantages over non-biodegradable membranes, including controlled resorption, elimination of secondary surgery, and the potential for delivery of bioactive agents. Preclinical studies in dogs have demonstrated that GBR membranes can promote periodontal regeneration, including bone and cementum formation and space maintenance; however, optimization of degradation behavior remains critical to align membrane resorption with tissue healing in some cases. Clinical studies in dogs with naturally occurring periodontal disease remain scarce, and only two biodegradable membranes (Ossiflex^®^ and Doxirobe^®^) are commercially approved for veterinary use, while all others are applied off-label. These limitations highlight the need for more adaptable and cost-effective regenerative strategies, including membranes that can be customized to different defect sizes and multifunctional membranes incorporating bioactive agents that will offer an advanced regenerative potential and improved predictability in veterinary periodontal therapy.

## 1. Introduction

In veterinary dental care, bone regeneration after periodontal disease in dogs remains one of the most complex challenges due to several factors. The main challenges include structural tissue loss, soft tissue competition, variable healing response, and chronic inflammation [1,2,3]. Disease progression is often also managed conservatively due to economic constraints, limited clinical evidence, or limited owner compliance with home care [1,4]. To address this challenge, guided bone regeneration (GBR) can be used, as it employs barrier membranes to direct the growth of bone and connective tissue while preventing soft tissue infiltration into the regenerating area [5]. Given their anatomical and clinical relevance, dogs are also the primary preclinical model for evaluating GBR membranes and studying periodontal regeneration. GBR membranes are applied after conventional periodontal treatment [4] and can be either non-biodegradable or biodegradable. However, from a clinical veterinary perspective, the use of GBR membranes in animals is restricted to biodegradable materials. Biodegradable GBR membranes can be produced from natural polymers, synthetic polymers, or a combination of both [6]. Additionally, biodegradable polymer matrices can be engineered to act as carriers for bioactive agents, enabling localized delivery to enhance bone regeneration and clinical outcomes [7]. The design of the membrane is essential, as it must meet the main criteria, detailed in Section 2, to ensure clinical success [8,9,10]. Recent studies aimed to improve the current gold standard of collagen membranes by exploring the use of other polymers [5,11]. Polymers play a crucial role in various applications, and advancements in technology continue to drive their exploration, as they are the foundation of many medical materials, including sutures, tubes, implants, and tissue engineering, for their versatility [12]. This review discusses the functional requirements of biodegradable GBR membranes, their performance in canines, and future directions in biodegradable membrane technology for periodontal regeneration in dogs.

## 2. Search Methodology

This is a narrative review of the literature on biodegradable membranes used in guided bone/tissue regeneration (GBR/GTR) for periodontal regeneration in dogs. A literature search was performed using Google Scholar and PubMed, using combinations of the terms “guided bone regeneration,” “guided tissue regeneration,” “biodegradable membrane,” “periodontal,” and “dog/canine,”. No restriction was applied regarding publication date; only articles published in English were considered.

Studies were included if they investigated the use of a biodegradable membrane for guided bone or tissue regeneration specifically in dogs, either in preclinical settings using surgically created periodontal or bone defects, or in clinical settings involving naturally occurring periodontal disease. Studies conducted in other animal models or human subjects, as well as those not involving a biodegradable membrane, were excluded. A small number of studies in other species were cited for background on membrane material properties but are not included in the tables summarizing dog-specific outcomes.

## 3. Functional Requirements of Biodegradable Membranes

Several types of membranes are used in GBR, including non-biodegradable membranes and biodegradable membranes. Non-biodegradable membranes require a second surgical procedure for removal, resulting in additional costs and increased surgical risks. In contrast, biodegradable membranes degrade naturally over time, allowing progressive material resorption during healing, and reduce complication rates and associated expenses [13,14]. Their biocompatibility, ability to provide temporary scaffolding for tissue repair, and prevention of soft tissue ingrowth have contributed to their increasing use in veterinary clinical practice, often achieving comparable or superior outcomes compared to non-biodegradable alternatives [11,15].

**Figure 1:**
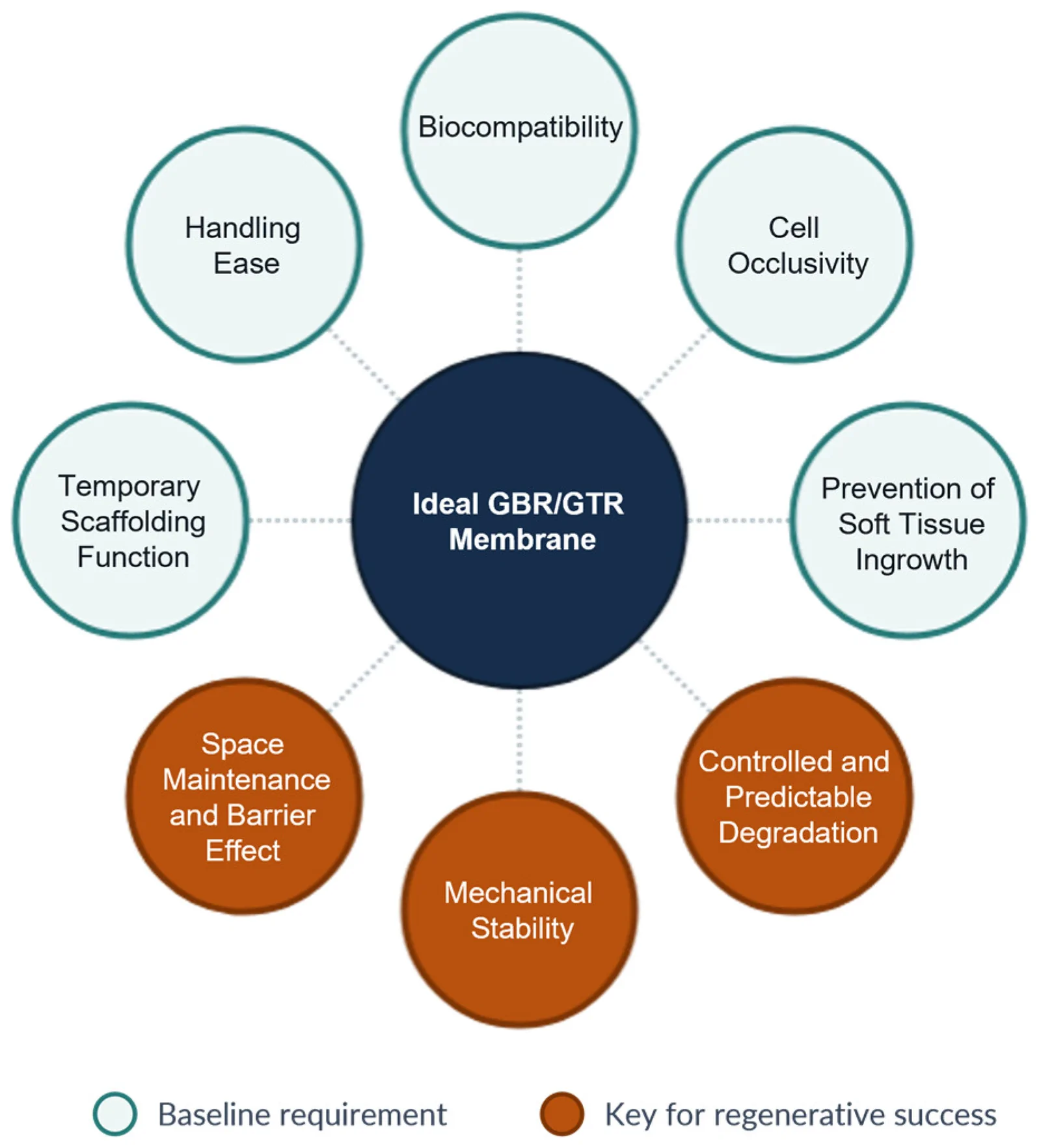
Ideal functional requirements of GBR/GTR membranes for tissue regeneration.

The degradation process of a biodegradable membrane plays a critical role in material performance and clinical success (Figure 1). Material degradation must occur in a predictable and biocompatible manner, preserving membrane morphology, mechanical stability, and barrier function during the early and intermediate phases of healing. Clinically, premature degradation may result in soft tissue infiltration and compromised bone formation, whereas excessively slow degradation can interfere with tissue remodeling and graft integration. Although ensuring long-term membrane stability remains challenging, promising results have been achieved by optimizing degradation rates to align with the tissue regeneration process and to retain the bone graft normally used with GBR membranes [7,16]. To modulate degradation kinetics and improve mechanical performance, crosslinking strategies are widely employed, particularly in natural polymer-based membranes. Crosslinking is a chemical or physical process that introduces inter- or intramolecular bonds between polymer chains, reducing polymer mobility, decreasing water uptake, and slowing enzymatic or hydrolytic degradation while enhancing mechanical strength. By adjusting crosslink density, membrane stiffness, durability, and resorption behavior can be tailored to meet both biological and clinical requirements [17,18,19,20].

Despite favorable clinical success rates in periodontal and bone regeneration, biodegradable GBR membranes still present material-related limitations, including variability in degradation behavior and reduced mechanical strength compared to non-biodegradable alternatives [10,11,21,22]. These challenges highlight the need for continued optimization of membrane composition and structure to improve both material performance and clinical predictability.

## 4. Biodegradable Guided Bone Regeneration Membranes in Canine

Guided bone and tissue regeneration (GBR/GTR) is considered an important therapeutic approach in canine periodontal therapy, supported by extensive pre-clinical research and an increasing, though still limited, number of clinical studies in dogs [8,19].

### 4.1. Pre-Clinical Studies

Canine models are well-established for preclinical evaluation of GBR membranes because of their anatomical, biological, and healing similarities to humans. Studies in dogs allow the assessment of key features of biodegradable membranes, such as safety and degradation [23]. The following sub-sections examine these material categories in detail and summarize the outcomes of studies conducted in canine models.

#### 4.1.1. Natural Polymers

Natural polymers are widely used in GBR membranes because of their favorable biological properties and multiple functional advantages. Collagen is the most widely used natural polymer in GBR membranes, while chitosan and bacterial cellulose also show significant potential (Table 1) [18,24,25,26]. As with all biodegradable membranes, degradation must be optimized to maintain barrier function long enough for bone regeneration while preventing soft tissue infiltration [19,20,27].

**Table 1:** Natural polymer membranes tested in canine pre-clinical studies.

Polymer	Description	Defect Type	Degradation	Commercially Available
Collagen	Non-cross-linked porcine types I and III collagen membrane	Acute peri-implant buccal dehiscence defects [19]; Chronic peri-implant buccal dehiscence defects [22,28]; Acute buccal periodontal dehiscence defects [18,29]; Acute alveolar extraction socket defects [11]; Acute critical-sized mandibular defects [19]; Acute alveolar ridge defects [21,30,31,32]; Chronic alveolar ridge defects [5,17,33]	N.D. [5,22,31]; Presence of the membrane: 3 weeks [33]; Light-to-moderate: 4 weeks [21,28]; Minor-to-complete degradation: 24 weeks [19]; Severe-to-complete: 4–8 weeks [17,32], 12 weeks [28,29], 16 weeks [10,19,21]; Complete degradation: 4 weeks [18], 12 weeks [11], 14 weeks [30]	Yes, Bio-Gide^®^ (Geistlich, Switzerland)
	Non-cross-linked porcine pericardium type I collagen membrane	Acute lateral alveolar ridge defects [32]	Severe-to-complete degradation: 8–12 weeks [32]	Yes, Remotis membrane (later Jason^®^ membrane; Botiss biomaterials, Germany)
		Acute square-shaped buccal periodontal dehiscence defects [34]	Complete degradation: 8 weeks [34]	Yes, Jason^®^ membrane (Botiss biomaterials, Germany)
				Yes, Smartbrane^®^ (Regedent, Switzerland)
	Dehydrothermally cross-linked porcine pericardium type I collagen membrane	Acute maxillary onlay bone grafting model [35]	Barrier effect: 12 weeks [35]	Yes, OssGuide^®^ (Hyundai Bioland, Republic of Korea)
	Diphenylphosphoryl azide cross-linked bovine type I collagen membrane with varying degradation rates	Acute horizontal circumferential periodontal defects [27]	Complete degradation: 3–4 months [27]	Yes, Paroguide^®^ (rapid-resorbing)
	Chemically cross-linked porcine collagen type I and III membrane	Acute alveolar ridge saddle-type defects [36]	Severe-to-complete degradation: 8 weeks [36]	No, Prototype VN (Geistlich Pharma, Switzerland)
	1-ethyl-3-[3-dimethylaminopropyl] carbodiimide (EDC) cross-linked porcine type I collagen membrane	Chronic narrow alveolar ridge defects [17]	Severe degradation: 4 weeks; Complete degradation: 8–16 weeks [17]	Yes, Collagen Membrane-P (Genoss, Suwon, Republic of Korea)
	1-ethyl-3-[3-dimethylaminopropyl] carbodiimide (EDC) cross-linked bovine type I collagen membrane	Acute peri-implant buccal dehiscence defects [24,37]; acute standardized buccal alveolar bone defects [38]	Severe-to-complete degradation: 8 weeks [24,37,38]	Yes, Collagen Membrane 1 Hard (Genoss, Suwon, Republic of Korea)
		Chronic peri-implant buccal dehiscence defects [39]	Moderate degradation: 16 weeks [39]	Yes, Collagen Membrane 2 Soft (Genoss, Suwon, Republic of Korea)
	Glutaraldehyde cross-linked bovine type I collagen membrane	Acute peri-implant buccal dehiscence defects [10]; acute buccal periodontal dehiscence defects [18]; acute mandibular one-walled periodontal defects [40]	Moderate degradation: 16 weeks [10]; Complete degradation: 4 weeks [18], 8 weeks [40]	Yes, BioMend^®^ (Zimmer Dental Inc, Carlsbad, CA, USA)
		Acute buccal periodontal dehiscence defects [18]	Complete degradation: 4 weeks [18]	Yes, Cytoplast RTM^®^ (CYT; Osteogenics Biomedical, Inc., USA)
	Enzymatic cross-linked porcine type I collagen membrane	Acute bilateral mandibular critical-size bone defects [19]	Barrier effect: 16 weeks; Severe to complete degradation: 24 weeks [19]	Yes, OSSIX^®^ Plus (ColBar LifeScience, Israel)
Chitosan + Collagen	1% and 2% chitosan-collagen membrane	Acute peri-implant buccal dehiscence defects [25]	Severe-to-complete degradation: 4 weeks [25]	No
Bacterial Cellulose	Electron beam-irradiated bacterial cellulose membrane	Acute peri-implant buccal dehiscence defects [24]	Presence of the membrane: 8 weeks [24]	No

##### Collagen

Non-cross-linked collagen membranes (Table 1) are normally derived from bovine or porcine sources and retain the native structure of collagen, which promotes cell adhesion and supports vascularization and tissue regeneration [17,33]. Several collagen membranes tested *in vivo* in canine models will be described below.

Remotis, Jason^®^ membrane, and Smartbrane^®^ are non-cross-linked porcine type I collagen membranes with reported effects on bone and cementum formation and a degradation time of eight to twelve weeks in dogs [32,34]. Remotis has an interconnected pore system that facilitates cell proliferation, demonstrates good biocompatibility both *in vitro* and *in vivo*, and supports tissue integration and vascularization. Compared with Bio-Gide^®^, it exhibited superior structural integrity and porosity in a canine buccal bone defect model [32]. In comparative analyses, Jason^®^ membrane showed superior cementum formation and bone regeneration compared to controls, while Smartbrane^®^ showed cementum formation comparable to 3D-printed gelatin membranes and increased bone formation relative to controls in canine critical-size dehiscence defects [34].

Bio-Gide^®^, a non-cross-linked porcine type I and III collagen membrane, is a bilayer membrane that can be used alone [21] or in combination with graft particles to stabilize them in dogs’ critical-size defects, chronic alveolar ridge defects, and two-wall chronified defects [5,19,33]. When combined with a poly-D, L-lactic acid (PDDLA) plaque, it resulted in greater tissue mineralization, likely due to its superior volume stability in saddle-type defects surgically created in dogs [30]. Both Bio-Gide^®^ and Remotis promote tissue integration, vascularization, and bone formation, although Remotis exhibits greater *in vivo* degradation resistance, persisting for eight to twelve weeks, compared with four to eight weeks for Bio-Gide^®^ in a canine buccal bone defect model [32]. Several studies suggest that regenerative outcomes improve when collagen membranes are combined with bone grafts [5,11,22,28,29], with results comparable to synthetic polylactic membranes [5,28], or even superior [28,29], although one study reports no added benefit over the graft alone [31]. Recent findings suggest limited regenerative outcomes at collagen-treated sites, with irregular ridge contours and fibrous tissue surrounding the graft in a canine alveolar bone defect model [11]. Overall, non-cross-linked collagen membranes provide the general benefits of collagen but exhibit inconsistent structural stability and variable regenerative performance [10,11,21,22].

Cross-linking slows collagen degradation and improves early mechanical stability. Because the barrier effect is essential, crosslinking strategies have been used to optimize the degradation and structural integrity of these collagen membranes [17,18,19,20]. 

OssGuide^®^, a dehydrothermally cross-linked membrane from porcine pericardium, maintained graft shape and barrier function during early healing in an alveolar ridge defect in a dog model, whereas Bio-Gide^®^ degraded rapidly within one to two weeks, showing early collapse. OssGuide^®^ provided superior dimensional stability in the initial period, but by eight to twelve weeks, both membranes supported comparable new bone formation [35]. To improve structural integrity and resorption time, chemical crosslinking agents such as diphenylphosphoryl azide (DPPA) and glutaraldehyde have been used [17,18,27]. DPPA-cross-linked rapid-resorbing membranes outperformed slow-resorbing ones and showed outcomes comparable to expanded Polytetrafluoroethylene (ePTFE) in horizontal circumferential defects created in dogs, whereas slow-resorbing membranes were associated with gingival recession and poor tissue integration [27]. Two dehiscences occurred with a prototype chemically cross-linked collagen membrane, leading to membrane exposure and affecting the eight-week outcomes. Although this prototype produced greater bone height in dog saddle-like defects than both the control (no membrane) and Bio-Gide^®^, the authors recommended further studies due to the 33.3% complication rate [36]. 

Genoss^®^ collagen membranes comprise three EDC-cross-linked type I collagen variants: Collagen Membrane 1 (Hard) and Collagen Membrane 2 (Soft), both bovine, and Collagen Membrane-P, porcine origin. Collagen Membrane 1 (Hard) has been shown to support bone regeneration and connective tissue formation in dog peri-implant dehiscence defects [24,37] as well as in standardized bone defects of healed edentulous ridges at eight weeks, but being severe to completely degraded at that time [38]. Collagen Membrane-P was compared with Bio-Gide^®^ to evaluate whether EDC crosslinking enhances tissue integration in a chronic narrow ridge. Although Bio-Gide^®^ degraded more rapidly, it showed superior integration, being replaced more quickly by dense connective tissue and displaying enhanced angiogenesis [17]. Notably, Collagen Membrane 2 (Soft), when combined with a bone graft, was significantly more favorable for horizontal bone regeneration of chronic buccal peri-implant dehiscence defects surgically created in dogs than Bio-Gide^®^ with a bone graft [39]. 

Cytoplast RTM^®^ and BioMend^®^, both glutaraldehyde-cross-linked bovine type I collagen membranes, implanted into dogs’ critical-size periodontal dehiscences, supported PDL regeneration with structural organization improving from four to eight weeks and were fully resorbed by four weeks. Cytoplast RTM^®^ achieved the greatest new bone height at four weeks, while Bio-Gide^®^ reached its peak in eight weeks. BioMend^®^ showed slower bone maturation [18] but supported new cementum, bone, and connective tissue formation at eight weeks, though chitosan-based membranes performed better in one-walled intrabony defects in dogs [40]. In another study, both Bio-Gide^®^ and BioMend^®^ promoted comparable bone regeneration in implant dehiscence defects, including increases in bone height, volume, and bone-to-implant contact by sixteen weeks. Though BioMend^®^ retained partial structural integrity slightly longer, both membranes’ outcomes were mainly influenced by space maintenance and avoidance of membrane exposure rather than degradation rate [10]. These differences highlight how degradation patterns and structural properties influence regeneration, while the cytotoxic potential of crosslinking agents remains a concern. As an alternative, enzymatic crosslinking via glycation, exemplified by GLYMATRIX^®^ technology, uses ribose to improve mechanical strength and degradation control [19]. OSSIX^®^ Plus, developed with GLYMATRIX^®^ technology, provided a barrier effect for up to sixteen weeks and nearly complete degradation at six months in dogs with bilateral critical-sized defects, with complete membrane ossification and no signs of inflammation, emphasizing its clinical potential [19].

##### Chitosan

Chitosan has been studied due to its biocompatibility, biodegradability, and antimicrobial properties [25]. These characteristics have motivated its investigation of membrane fabrication in bone regeneration due to its ability to support cell growth and tissue repair and enhanced physicochemical properties, especially when cross-linked with β-glycerol phosphate [41]. In veterinary research, chitosan-collagen membranes were evaluated as more stable alternatives to collagen alone. Although 2% chitosan-collagen membranes improved vertical bone growth in canine acute buccal dehiscence-type defects compared with no treatment, neither formulation outperformed the collagen membrane, likely due to degradation at four weeks that impaired the barrier effect and limited bone formation [25].

##### Cellulose

Bacterial cellulose exhibits good biocompatibility and physicochemical properties, resembling collagen membranes, and forms a highly pure nanofibrous network [25]. However, its low biodegradability, slow degradation rate [42], and limited cellular response restrict its use in GBR. To enhance resorption and cell adhesion, electron beam irradiation has been used to increase membrane porosity while maintaining tensile strength. Electron beam-irradiated bacterial cellulose achieved cell adhesion, proliferation, and bone regeneration comparable to a commercial collagen membrane (Collagen Membrane 1, Genoss) in canine peri-implant dehiscence defects after eight weeks [24].

#### 4.1.2. Synthetic Polymers

Synthetic polymers include polylactic acid (PLA), polyglycolic acid (PGA), polycaprolactone (PCL), and their copolymers such as poly(lactic-co-glycolic acid) (PLGA) and poly(lactide-co-caprolactone) (PLCL), poly-lactic acid-co-glycolic acid-co-ε-caprolactone (PLGC), as well as hydrophilic polymers like polyethylene glycol (PEG). These materials are widely used in biomedical applications due to their reproducible processing and customizable properties. By adjusting polymer composition, monomer ratios, or incorporating PEG segments, degradation rate, mechanical behavior, and hydrophilicity can be customized to match the needs of tissue regeneration [43].

##### Polylactic Acid (PLA)

Guidor^®^, a double-layer polylactic acid-citric acid ester membrane, supported new bone formation, revascularization, and later bone maturation, performing similarly to Goretex^®^ and enhancing tissue ingrowth compared with untreated alveolar ridge defects [15]. When combined with bone grafts, it improved early outcomes and achieved hard- and soft-tissue regeneration in two-wall chronified bone defects in dogs comparable to the collagen membrane Bio-Gide^®^ [5]. In peri-implant dehiscence defects, Guidor^®^ and Bio-Gide^®^ both facilitated new bone formation with or without grafts. Guidor^®^ provided better space maintenance and greater tissue width for four weeks. Though volume loss was observed in the Guidor^®^ group by twelve weeks, differences were not significant. Notably, Guidor^®^ alone still produced substantial bone at both time points, with the membrane-only group yielding more new bone than groups treated with a graft in peri-implant dehiscence defects [28]. Complete to moderate degradation was reported after three months in all the studies (Table 2) [5,15,28].

PLA materials differ in degradation profiles, with crystalline Poly-L-lactic acid (PLLA) and poly-D-lactic acid (PDLA) degrading slowly, while amorphous poly-D, L-lactic acid (PDLLA) degrades more rapidly [44]. Early DL-PLA membranes effectively regenerated bone, cementum, and connective tissue in buccal periodontal defects created in dogs, with degradation rates adjustable by altering oligomer content. Incorporating higher proportions of low-molecular-weight PLA oligomers accelerated degradation, while lower proportions slowed it down. These findings allow membranes to be designed for the specific requirements of a given defect [16]. ATRISORB^®^, composed of DL-PLA dissolved in N-methyl-2-pyrrolidone (NMP), solidifies on contact with moisture and can be molded directly onto defects. It has shown biocompatibility and controlled degradation in canine models, demonstrating regeneration of bone, cementum, and periodontal ligament in both class II furcation defects [45] and naturally occurring buccal dehiscence defects in dogs [46].

**Table 2:** Synthetic polymer membranes in canine pre-clinical studies.

Polymer	Description	Defect Type	Degradation	Commercially Available
Polylactic Acid (PLA)	Double-layer PLA blended with acetyl tributyl citrate membrane	Acute standardized through-and-through alveolar bone defects [15]; Class III Periodontal furcation defects [47]	Fragmented and replaced by a granuloma at 6 months [47]; Complete degradation: 3 months [15]	Yes, Guidor^®^ (Guidor AB, Sweden)
		Chronic peri-implant buccal dehiscence defects [5,28]	No degradation to minor degradation: 4 weeks; Moderate degradation: 12 weeks [5,28]	Yes, GUIDOR^®^ (Sunstar, Switzerland)
	Thin films of high molecular weight (Mw) PLA with added low-Mw oligomers (0–30%)	Acute buccal periodontal dehiscence defects [16]	Complete degradation: 0% and 10%—More than 6 months; 30%—3 to 4 months [16]	No
	DL-PLA dissolved in N-methyl-2-pyrrolidone (NMP) composite membrane	Class II periodontal furcation defects [45];	Suggested 9 to 12 months until full absorption; Barrier present at 6 months [45];	Yes, Atrisorb^®^ (Birmingham Polymers/Atrix Laboratories, USA)
		Chronic periodontal dehiscence defects [46]	Light degradation: 5 months; Marked degradation: 6 to 8 months [46]	
Poly(lactic-co-glycolic acid) (PLGA)	Polylactide-glycolide copolymer film	Class II periodontal furcation defects [48]; Class III periodontal furcation defects [47,49,50].	N.D. [47,48,49,50].	Yes, Gore Resolut^®^ (Gore-Tex, USA)
	Double-layer PLGA membrane	Acute alveolar ridge defects [7]	5–6 months advertised by the manufacturer; Moderate degradation: 4 months [7]	Yes, Gore Resolut Adapt membrane^®^ (W.L. Gore & Assoc., USA)
	Poly-d,l-lactide-co-glycolide membrane	Acute alveolar extraction socket defects [51]	Complete degradation: 4 weeks [51]	Yes, supplied by Chongqing Yongtong BIO-Pharmaceutical Limited Liability Co., Ltd.
		Acute alveolar extraction socket defects [11]; Acute created osseous periodontal defects [52]	Complete degradation (advertised): 16 weeks; N.D. [11]; Severe degradation: 12 weeks [52]	Yes, GC Membrane (GC Co., Tokyo, Japan)
	Electrospun PLGA fibers (50:50 PLA/PGA)	Acute V-shaped buccal periodontal dehiscence defects [29]	Severe degradation: 12 weeks [29]	No
Poly(lactic acid/caprolactone) (PLCL)	Poly(lactic acid/caprolactone) bilayer membrane	Acute alveolar extraction socket defects [11]	N.D. [11]	No
Poly-lactic acid-co-glycolic acid-co-ε-caprolactone (PLGC)	Macroporous PLGC membrane combined with a collagen sponge embedded in basic fibroblast growth factor	Acute alveolar ridge defects [53]	Marked degradation: 6 months [53]	No
Polyethylene glycol (PEG)	Cross-linked PEG-based gel for in situ-created membrane	Chronic peri-implant buccal dehiscence defects [54]; Acute alveolar ridge defects [7]	N.D. [54]; Minor degradation: 4 weeks; Marked to complete degradation: 4 months [7]	Yes, PEG membrane (Institut Straumann AG, Switzerland)

##### Poly(Lactic-Co-Glycolic Acid) (PLGA)

Gore Resolut^®^, a PLGA membrane, showed outcomes comparable to non-resorbable e-PTFE membranes in promoting cementum and bone formation in class III furcation defects surgically created in dogs [49] and outperformed Guidor^®^ at six months by enhancing bone and periodontal ligament formation in class III furcation defects, whereas granuloma formation limited healing in Guidor^®^-treated sites [47]. When combined with cell sheets, it further enhanced periodontal regeneration in class II and III defects, particularly new bone formation [48,50]. Its successor, Gore Resolut Adapt^®^, incorporates polyglycolic acid/trimethylene carbonate (PGA-TMC) to improve flexibility and handling while retaining barrier and degradation characteristics, supporting strong bone formation, graft containment, and moderate degradation at four months in acute standardized defects created in dogs [7].

Other PLGA-based membranes show variable performance. For example, one matched collagen membrane in bone formation in extraction sockets in dogs but fully degraded within four weeks [51]. The PGA/PLA-based GC Membrane degraded more slowly, with residual fragments at twelve weeks, membrane collapse, incomplete bone formation, and an irregular ridge with fibrous tissue surrounding the graft, although overall bone formation remained comparable to Bio-Gide^®^ in alveolar bone defects in dogs [11,52]. An electrospun 50:50 PLGA membrane combined with bone substitutes showed partial degradation and limited bone formation in a V-shaped buccal dehiscence in dogs, with Bio-Gide^®^ performing better [29].

##### Poly(Caprolactone) (PCL)

Copolymerization of PCL with PLA or PLGA has been used to develop membranes with controlled degradation and improved functional properties [11,53]. Poly(lactic acid/caprolactone) (PLCL), a bilayer copolymer membrane incorporating ε-caprolactone into PLA, exhibits slower and more predictable resorption. Its compact layer provides an effective barrier against bacterial infiltration [55], while the porous layer supports cell proliferation and differentiation *in vitro*. In an alveolar bone defect model surgically created in dogs, PLCL membranes maintained regular alveolar ridges and promoted marrow and cortical bone regeneration [11]. Similarly, in lateral bone defects in dogs, poly(lactic-co-glycolic acid-co-ε-caprolactone) (PLGC) membranes combined with collagen sponges and growth factor allowed connective tissue infiltration, stabilized the membrane, and enhanced vascularization within and around the pores, although the independent effect of the membrane was not fully assessed [53].

##### Polyethylene Glycol (PEG)

PEG membranes placed over grafted bone defects in dogs were found to be cell-occlusive, allowing only macrophage penetration, whereas collagen membranes allowed infiltration of both fibrocytes and macrophages [54]. Jung et al. suggested that this reduced soft-tissue integration could be a potential drawback. Although this limited soft-tissue integration was noted as a potential drawback, PEG produced similar amounts of new bone as collagen (Bio-Gide^®^) and showed no significant advantage over grafts alone after six months [54]. Comparable outcomes were also reported when PEG was compared with a double-layer PLGA membrane (Resolut Adapt membrane^®^) regarding new bone formation and containment of the bone graft in acute standardized defects created in dogs [7].

#### 4.1.3. Combination of Natural and Synthetic Polymers

Blending natural and synthetic polymers is a common strategy to merge the biological advantages of natural polymers with the mechanical strength and controlled degradation of synthetic polymers. Such hybrid systems have been investigated in preclinical studies in dogs, but their performance has been limited, with unpredictable degradation, inflammation, and inconsistent support of bone formation [56,57].

##### Polyhydroxybutyrate-Hydroxyvalerate (PHB-HV) and Polyglactin 910

Polyhydroxybutyrate-hydroxyvalerate (PHB-HV), a bacterial polymer, reinforced with polyglactin 910 to form the PHB-HVIPG membrane, showed minimal bone filling and induced inflammation when applied immediately after implant placement in dogs. Its unpredictable degradation (Table 3) raised concerns about its suitability for GBR membrane production [56].

##### PLA and Collagen

Hybrid membranes combining poly(lactic acid) (PLA) with collagen faced similar challenges. In dogs, two intercalated collagen membranes with a PLA layer failed to support bone regeneration, as PLA induced a foreign body reaction and inflammation, increasing resorption of newly formed bone in lateral bone defects in dogs [57].

**Table 3:** Natural and synthetic polymer composite membranes in canine pre-clinical studies.

Polymers	Description	Defect Type	Degradation	Commercially Available
Polyhydroxybutyrate-hydroxyvalerate reinforced with polyglactin 910 (PHB-HV/PG)	Hydrolyzable polyester material	Acute alveolar extraction socket peri-implant defects [56]	N.D. [56]	No
Collagen and polylactide (PLA)	Membrane is composed of 3 layers: Two of collagen and one of polylactide (PLA)	Chronic healed mandibular lateral alveolar ridge defects [57]	Complete degradation (collagen): 4.5 months; Moderate degradation (PLA): 4.5 months [57]	No

#### 4.1.4. Multifunctional Membranes

GTR membranes have traditionally functioned as passive barriers, preventing soft tissue invasion and preserving space for bone healing. However, many conventional membranes fail to meet the complex biological and mechanical demands of bone regeneration [58]. To overcome these limitations, multifunctional membranes (Table 4) have been developed, combining barrier function with properties such as antimicrobial activity, osteoconductivity, angiogenic stimulation, or controlled release of bioactive molecules. Incorporating bioactive agents, solvents, or coatings with natural ceramics or clays can further enhance regenerative potential [29,40,52,59].

**Table 4:** Multifunctional polymer membranes in pre-clinical canine studies.

Type of Bioactive Agent	Polymer	Description	Defect Type	Degradation	Commercially Available
Antibiotics	Polylactic-co-glycolic acid (PLGA)	PGA and PLA copolymerized GTR membrane (GC membrane) loaded with 25% doxycycline	Acute created osseous periodontal defects [52]	Severe degradation: 12 weeks [52]	No
		Metronidazole-loaded PLGA films produced by the solvent-casting method	Acute buccal periodontal dehiscence defects [60]	N.D. [60]	No
Polyphenolic Compound + Statin Drug	Chitosan	Three-layered chitosan membrane loaded with a natural catechin, epigallocatechin-3-galate (EGCG), and a statin, lovastatin	Acute mandibular one-walled periodontal defects [40]	Complete degradation: 8 weeks [40]	No
Ceramics	Polyhydroxybutyrate (PHB)	Rigid PHB membranes containing 25 to 35% hydroxyapatite	Class II periodontal furcation defects [61]	N.D. [61]	No
	Poly-L-lactide	β-tricalcium phosphate combined with copolymerized poly-L-lactide by the heat kneading method	Acute critical-sized mandibular defects [59]	Present: 4 weeks; Complete degradation: 8 weeks [59]	No
	Poly(l-lactide-co-glycolide-co-e-caprolactone) (PLGC)	β-tricalcium phosphate combined with copolymerized poly(l-lactide-co-glycolide-co-e-caprolactone) by heat kneading method	Acute critical-sized mandibular segmental bone defects [62]	In PBS, 24 weeks; *In vivo*, presence is not clear at 4 weeks [62]	No
	Collagen and Chitosan	Fish collagen, chitosan, and bioactive glass (Col/BG/CS)	Class II periodontal furcation defects [63]	N.D. [63]	No
	Collagen and poly(L-lactide)	Composite of nano-hydroxyapatite combined with a natural (collagen) and a synthetic (PLA) polymer	Ectopic subcutaneous implantation model [64]	Collagen degradation after 2 weeks of implantation [64]	No
Clay	PLGA	Electrospun attapulgite (ATT)-doped PLGA scaffold	Acute V-shaped buccal periodontal dehiscence defects [29]	Severe degradation: 12 weeks [29]	No
Solvent	PLGA	Poly(lactide-co-glycolide) trimethylene carbonate-based and N-methyl pyrrolidone (NMP)-releasing membrane (PLGA/NMP)	Acute peri-implant buccal dehiscence defects [65]	PLGA/NMP membranes: Severe degradation: 3 months; PLGA/NMP membranes + graft: Intact: 3 months [65]	Yes, Inion^®^ GTR™ (Inion Oy, Finland)
Growth factor	Poly(lactide-co-glycolide)	Construct of PLGA combined with recombinant human growth/differentiation factor-5 (rhGDF-5/PLGA)	Acute standardized buccal periodontal pocket defects [66]	Moderate degradation: 2, 4, and 6 weeks [66]	No

##### Antibiotics

Tetracyclines are notable for their dual antibacterial and regenerative effects. Topical doxycycline, for example, enhances osteogenesis and, when combined with a PLGA membrane, prevents infection and promotes new attachment and bone formation compared to PLGA alone, although membrane collapse into the defect and severe degradation (Table 4) were observed at twelve weeks in alveolar bone defects in dogs [52]. In contrast, metronidazole-loaded membranes did not show additional benefits over PLGA alone, despite promoting some new attachment and bone formation compared to untreated buccal dehiscence-type defects in dogs [60].

##### Polyphenols

Polyphenols are plant-derived compounds with antioxidant, anti-inflammatory, and antimicrobial properties, making them attractive for incorporation into biomaterials to support tissue regeneration and prevent infection [67]. A trilayer chitosan membrane, inherently antibacterial, was developed with epigallocatechin-3-gallate (EGCG) on the surface and controlled release of lovastatin from the middle layer. When used with a hemostatic gelatin-based sponge and compared to the collagen membrane BioMend^®^, it enhanced the formation of new cementum, bone, and connective tissue and showed enhanced bone regeneration in one-walled intrabony defects created in dogs [40].

##### Natural Ceramics

Natural ceramics such as hydroxyapatite, β-tricalcium phosphate (β-TCP), and bioactive glass are incorporated into membranes for their biocompatibility and osteoconductive properties, providing scaffolds for cell attachment and proliferation [61,62,63]. Attempts to integrate ceramics into membranes included polyhydroxybutyrate (PHB) membranes containing 25–35% hydroxyapatite. However, these rigid membranes showed limited bone, cementum, and periodontal ligament regeneration in dog class II furcation defects and were associated with inflammatory infiltration. Early membrane exposure from day eight and the lack of adaptability of the rigid membrane to the defect likely contributed to these results, as contamination normally impairs bone regeneration [61].

More adaptable ceramic-polymer composites incorporating β-TCP were developed. A copolymerized poly-L-lactide/β-TCP membrane (TCP/CPLA) demonstrated significant bone regeneration in surgically created mandibular defects in dogs, producing 90% new bone fill at twelve weeks, although the membrane was not radiographically visible at eight weeks [59]. Due to the high softening temperature and potential cytotoxicity of the TCP/CPLA membrane, a modified composite incorporating β-TCP and poly(L-lactide-co-glycolide-co-ε-caprolactone) (PLGC) was later introduced. This more flexible membrane generated significant bone regeneration, achieving a 95% increase in bone height compared to 5% in controls at twelve weeks, though it was radiographically undetectable at four weeks [62].

Based on the need for improved membrane adaptability and biological performance, combining ceramics with polymer blends has emerged as a strategy to better mimic bone architecture and enhance structural integrity and cellular interactions [64]. Such composites can include natural-natural, synthetic-synthetic, or natural-synthetic polymer blends, often integrated with ceramics to improve biocompatibility, osteoconductivity, and mechanical stability [37,63,64]. A representative natural-natural composite is the fish collagen/chitosan/bioactive glass (Col/BG/CS) membrane. In a dog class II furcation defect model, this membrane enhanced new bone formation, promoted dense connective tissue, and reduced inflammation compared to control defects at both one and two months. It also showed *in vitro* antibacterial activity against *Streptococcus mutans* [63]. For natural-synthetic blends, a nano-hydroxyapatite/collagen/poly(L-lactide) (nHAC/PLA) composite supported the implantation of osteogenic-induced periodontal ligament stem cells in subcutaneous pockets, demonstrating biocompatibility and promoting cell proliferation. Although collagen degraded after two weeks, the nano-hydroxyapatite and PLA matrix remained intact. This composite also produced greater new bone formation compared to a hydroxyapatite/tricalcium phosphate (HA/TCP) graft [64].

##### Clay

Clays are emerging additives in GBR membranes due to their biocompatibility and functional properties. Attapulgite, a magnesium-aluminum phyllosilicate, provides a porous scaffold and releases magnesium and silicon ions that stimulate osteoblast activity, collagen synthesis, and hydroxyapatite formation [68]. When incorporated into an electrospun PLGA membrane, attapulgite enhanced bone regeneration to levels comparable with Bio-Gide^®^ and better than PLGA alone in V-shaped buccal dehiscence in dogs [29].

##### Solvent

Solvents can act as bioactive agents by increasing permeability, supporting controlled release, and influencing biological processes. A PLGA/NMP membrane showed performance comparable to the collagen membrane Bio-Gide^®^ when used with a bone substitute and tended to increase horizontal bone formation in buccal dehiscence defects. However, without a bone graft, the PLGA/NMP membrane performed worse than Bio-Gide^®^, indicating the need for graft material to achieve favorable outcomes [65].

##### Growth Factors

Growth factors can be incorporated into GBR membranes to promote osteogenesis. In a defect model, an injectable poly(lactide-co-glycolide) (PLGA) composite combined with recombinant human growth/differentiation factor-5 (rhGDF-5) showed controlled degradation and enhanced bone formation in surgically created periodontal pockets over the buccal roots compared with the control [66].

### 4.2. Clinical Studies in Dogs with Naturally Occurring Periodontal Disease

While pre-clinical research in dogs based on surgically created defects is abundant in the literature, there is a notable scarcity of large-scale clinical trials focusing on dogs with naturally occurring periodontal disease [8,14,69,70]. This distinction is critical because surgically induced defects may not fully replicate the complexity and dynamics of the microenvironment found in dogs [69].

In this context, although the use of membranes for GBR/GTR is well established and moderately common in specialized veterinary clinics, their broader clinical application remains limited. In everyday decision-making, their application depends on factors such as defect type, surgical skill, cost, and the owner’s interest in preserving strategically important teeth [8,71]. Membranes are more frequently employed in vertical periodontal defects and in the management of strategic teeth, such as canines and premolars, where tooth preservation is a priority [72,73], whereas they are less commonly used in extensive horizontal defects or in very small or brachycephalic dogs, where the technique may be more challenging [69].

Nevertheless, recent clinical studies have provided data on the performance of various bioabsorbable membranes in dogs [8]. Among these, only a limited number of membranes are currently validated for veterinary use, namely a demineralized freeze-dried bone membrane allograft (DFBMA; Ossiflex^®^) [74,75] and a doxycycline-containing liquid polymer, Doxirobe^®^ [71,76,77], in contrast to other membranes that have been used as off-label. Ossiflex^®^ is made from canine cortical bone and, to our knowledge, is the only commercially available barrier membrane specifically validated for veterinary use. Clinical evaluations of intrabony periodontal defects have shown that DFBMA used in combination with bone allografts can reduce probing depths and restore physiological values at treated sites, with similar improvements in periodontal probing reported in infrabony defects [72,75]. In a large retrospective analysis of 112 procedures, bone membranes were significantly more effective than liquid polymer barriers in achieving attachment gain, particularly in deep vertical defects [8]. Despite these differences, the liquid polymer membrane Doxirobe^®^ remains an important option in cases where adaptability to irregular defect morphology is critical, offering advantages in handling and defect conformity that solid membranes cannot provide. Doxirobe^®^ is composed of N-methyl-2-pyrrolidone and poly(DL-lactide) containing 8.5% doxycycline and is approved for local antibiotic delivery in veterinary medicine but is commonly used for GTR as an off-label product [71,76]. The main clinical advantage lies in handling characteristics, as the material is initially liquid and can adapt closely to irregular defect morphology before polymerizing into a solid barrier. Case series report reductions in probing depth and radiographic evidence of new alveolar bone formation at six months [76]. However, due to their tendency to slump, these materials may collapse if not supported by bone grafts. When added in combination with an autogenous cortical graft [71] or bioactive glass particles [77] has shown promising results, particularly in vertical defects.

Beyond membranes validated for veterinary use, several off-label products adapted from human dentistry have been studied for canine GTR/GBR in dogs with naturally occurring PD disease. Collagen membranes with different degrees of cross-linking, Paroguide^®^ and Colética (Colética Corp., Lyon, France), were compared to the non-resorbable ePTFE membrane, Goretex^®^, achieving full regenerative effect by four weeks, while ePTFE required up to twelve weeks, likely due to removal at six weeks. All three membranes, weakly cross-linked collagen, strongly cross-linked collagen, and non-resorbable ePTFE, produced comparable levels of new attachment, bone, and cementum in naturally occurring periodontal defects despite variations in degradation rates [14]. In addition, polycaprolactone, polycarbonate, silicone rubber, and ePTFE membranes demonstrated similar promotion of new cementum and bone in class II furcation defects in dogs, with regeneration significantly exceeding that of untreated controls. Some synthetic membranes, such as polycaprolactone and polycarbonate, triggered higher inflammatory responses, likely due to slower degradation, while silicone rubber induced minimal inflammation. No single membrane showed clear superiority, and complete regeneration was not achieved [70]. Guidor^®^, a double-layer polylactic acid-citric acid ester membrane, has demonstrated effective periodontal and bone regeneration in naturally occurring buccal class II furcation defects. It outperformed e-PTFE (Goretex^®^) in promoting periodontal regeneration and reducing inflammation, and showed results comparable to polydioxanone membranes, which supported new bone, cementum, and periodontal ligament formation, with both materials achieving outcomes similar to a healthy furcation by twelve months [9]. These findings reinforce that bioresorbable membranes can effectively replace non-resorbable barriers, achieving similar regenerative outcomes while allowing less invasive treatment and eliminating the need for a second surgery, though further comparative studies are needed to optimize clinical application.

Keeping this in mind, recent strategies have explored bioresorbable scaffolds, such as ReGum™, a porcine gelatin matrix cross-linked with transglutaminase, as a porous scaffold for periodontal regeneration. In a multicenter clinical study involving dogs with naturally occurring periodontitis, the addition of this implant to open periodontal therapy resulted in significant improvements in probing depth reduction compared with root planing only. Gelatin, a derivative of collagen, allows for preserving the cellular binding sites that promote tissue regeneration, and these scaffolds were reported as safe, easy to handle, and particularly effective in increasing alveolar bone height on the buccal aspect [69]. Although these membranes are not currently licensed for clinical use in dogs, their evaluation and approval for veterinary use are important to ensure safety and predictable clinical outcomes.

### 4.3. Cross-Study Synthesis and Comparative Analysis

Across the membrane categories reviewed above, degradation rate alone did not consistently predict the regenerative outcomes, both in preclinical and clinical settings. Within collagen membranes, faster-degrading formulations such as Bio-Gide^®^ matched or outperformed more resistant cross-linked alternatives [17,35], while slow-resorbing DPPA cross-linked membranes underperformed rapid-resorbing ones [27]. Synthetic membranes showed a similar pattern, where the predictability of the degradation and not the degradation rate distinguished the PLGA formulations with better outcomes from the ones associated with membrane collapse [11,51,52]. PEG membranes further demonstrated that properties independent of degradation, such as cell-occlusivity, can limit outcomes despite resorption times comparable to collagen [7,54]. The combination of natural and synthetic showed no controllable degradation, with unpredictable resorption of the synthetic polymer compromising outcomes despite the natural polymer biocompatibility [56,57]. Clinical studies showed results similar to the preclinical ones, with membranes with different degradation profiles demonstrating comparable attachment, bone, and cementum formation [9,14,70].

Structural stability, space maintenance, and adaptability to defect morphology instead predicted more consistently the regenerative success of the membranes. Comparable outcomes between BioMend^®^ and Bio-Gide^®^ were attributed to space maintenance and avoidance of membrane exposure and not to degradation rate [10]. Regarding multifunctional membranes, mechanical adaptability, rather than the incorporated bioactive agent, determined whether the additional functionality translated into improved performance. For example, rigid ceramic-loaded membranes failed despite their osteoconductive potential [61], whereas more flexible ceramic-polymer composites achieved substantial bone regeneration [59,62]. In clinical settings, handling and adaptability to the irregular defect morphology were more relevant than degradation rate, allowing the use of Doxirobe^®^ despite bone membrane allografts achieving greater attachment gain in deep vertical defects [8].

**Figure 2:**
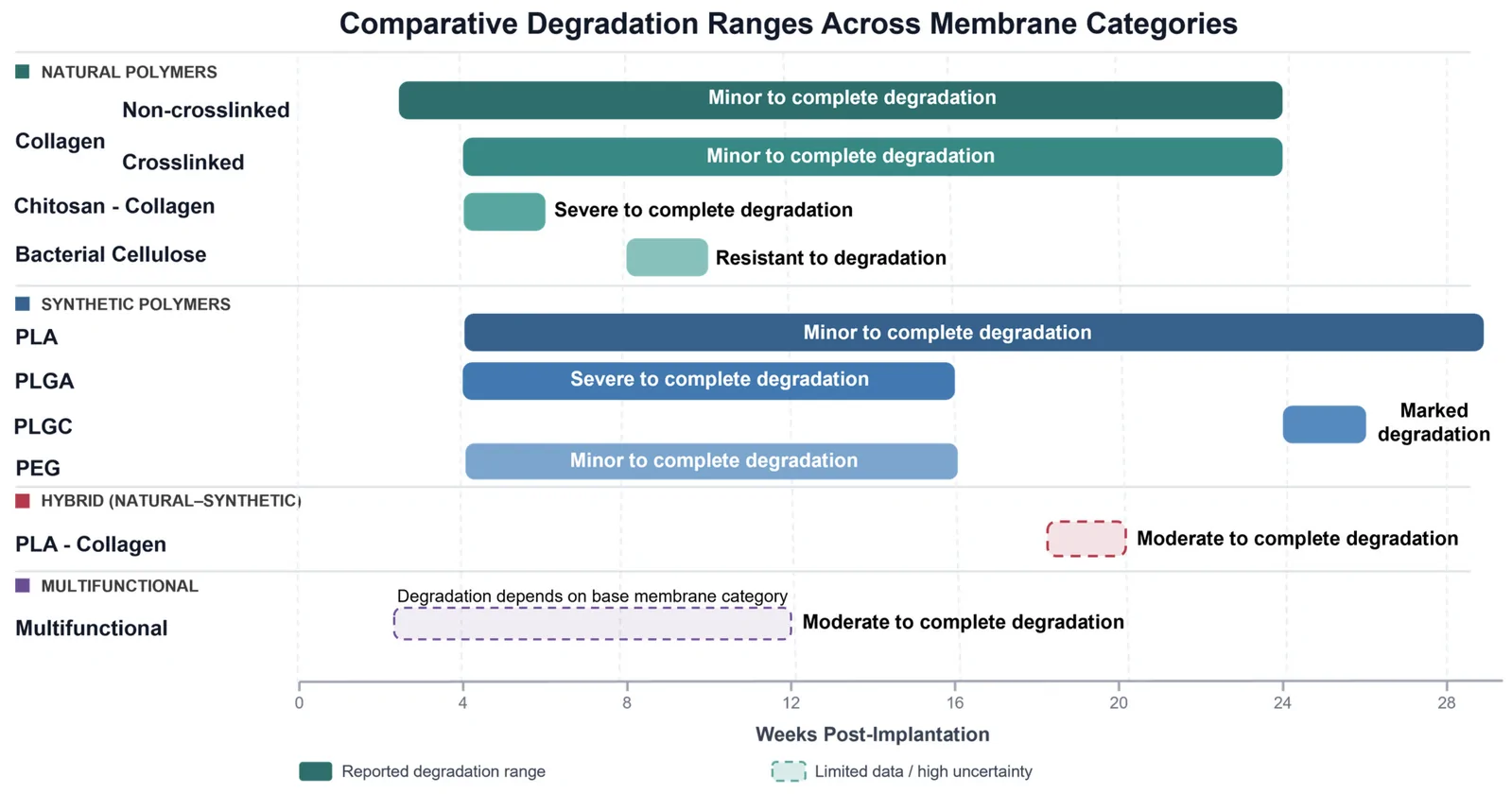
Comparative degradation ranges of biodegradable membrane categories evaluated in canine GBR/GTR studies. Ranges reflect reported degradation data across included studies. The variation within each category results from differences in defect model and healing period across the underlying studies.

Comparing across categories (Table 5, Figure 2), natural polymers offer strong biocompatibility and handling but variable barrier maintenance, which can go from early collapse [35] to a sixteen-week barrier effect [19] depending on the cross-linking used. Synthetic polymers allow more reproducible, customizable degradation [16], though poorly controlled formulations still result in collapse and irregular ridge contour [11,52]. Hybrid natural-synthetic membranes perform least favorably overall, with unpredictable degradation and inflammation limiting bone formation regardless of material combination [56,57]. While multifunctional membranes have an increased potential by allowing the incorporation of a wide range of biological functions, their success is determined more by their structural properties rather than the incorporated bioactive agent [52,61,62,65]. Complications, including membrane exposure, foreign body reaction, and collapse, occurred across all four categories, not being limited by a single material type [11,27,36,56,57,61]. Therefore, structural stability, space maintenance, and adaptability to defect morphology emerge as more reliable predictors of regenerative success than degradation rate or material type, both in preclinical and clinical settings in dogs.

## 5. Future Perspectives in Biodegradable Membrane Technology for Canine Periodontal Regeneration

Future directions in biodegradable membrane technology for canine periodontal regeneration are increasingly centered on the development of multifunctional membranes capable of combining physical barrier function with additional biological and mechanical roles, while allowing better adaptation to the wide variability of periodontal defects seen in dogs. 3D printing is gaining attention as a manufacturing strategy for controlling membrane architecture, mechanical behavior, and degradation kinetics, with the added advantage of customization to defect size and shape [34,78,79]. Experimental studies using 3D-printed membranes made from natural or composite polymers have shown regenerative outcomes comparable to, and in some cases superior to, conventional collagen membranes, supporting bone and cementum formation and maintaining space for regeneration [34,37,79].

Despite these advances, the clinical use of GBR/GTR in dogs remains highly case-dependent. Practical limitations such as increased cost, technical complexity, longer surgical and anesthesia times [10], and the small number of membranes validated specifically for veterinary use continue to restrict their broader use [8]. In addition, not all periodontal defects are suitable for regenerative therapy, as small or predominantly horizontal lesions often show limited benefits. Clinical outcomes are strongly influenced by defect morphology, surgical technique, and owner compliance, while non-resorbable membranes further reduce feasibility due to the need for a second surgical procedure [8,69,74,75]. Even so, when cases involve vertical defects and the preservation of strategic teeth, GBR/GTR remains a valid alternative to extraction [8,77].

Overall, these constraints point to the need for new, more adaptable regenerative strategies and for their clinical testing in dogs with naturally occurring periodontal disease. In this regard, advanced biodegradable membranes produced through 3D printing offer a dual advantage: customization to individual defect morphology and the ability to tailor key material properties through controlled scaffold design, which may help overcome current limitations and improve the predictability and clinical relevance of regenerative periodontal therapy in veterinary practice.

## Figures and Tables

**Table 5:** Comparative overview of biodegradable membrane categories evaluated in canine GBR/GTR studies.

Membrane Category	Barrier Maintenance	Handling	Degradation Controllability	Mechanical Strength	Complication Rate
Natural polymers	Variable ranging from early collapse (1–2 weeks) to prolonged barrier function (16 weeks), depending on cross-linking [19,35]	Good	Low to moderate	Low to moderate	Moderate due to membrane exposure and/or early collapse
Synthetic polymers	Customizable through polymer composition and monomer ratio	Moderate to good	High being adjusted by design [16]	Moderate to high	Variable depending on formulation [11,52]
Combination of natural and synthetic	Poor	Poor	Low	Low to moderate	High with reports of inflammation and foreign body reaction
Multifunctional	Dependent on the base membrane	Variable	Dependent on the base membrane	Dependent on the base membrane	Moderate and dependent on adaptability of the base membrane

## Data Availability

No new data were created or analyzed in this study. Data sharing is not applicable to this article.

## References

[1] Pinho L.C., Santos C., Fernandes M.H., Colaço B. (2025). Canine periodontal ligament stem cells as a tool for periodontal regeneration. Res. Vet. Sci..

[2] Cunha E., Tavares L., Oliveira M. (2022). Revisiting Periodontal Disease in Dogs: How to Manage This New Old Problem?. Antibiotics.

[3] Wallis C., Holcombe L.J. (2020). A review of the frequency and impact of periodontal disease in dogs. J. Small Anim. Pract..

[4] Niemiec B.A. (2008). Periodontal Therapy. Top. Companion Anim. Med..

[5] Di Raimondo R., Sanz-Esporrín J., Sanz-Martin I., Plá R., Luengo F., Vignoletti F., Nuñez J., Sanz M. (2021). Hard and soft tissue changes after guided bone regeneration using two different barrier membranes: An experimental in vivo investigation. Clin. Oral Investig..

[6] Broda M., Yelle D.J., Serwańska-Leja K. (2024). Biodegradable Polymers in Veterinary Medicine—A Review. Molecules.

[7] Thoma D.S., Dard M.M., Hälg G.A., Ramel C.F., Hämmerle C.H.F., Jung R.E. (2012). Evaluation of a biodegradable synthetic hydrogel used as a guided bone regeneration membrane: An experimental study in dogs. Clin. Oral Implant. Res..

[8] Lee B.L., Soukup J., Rendahl A., Goldschmidt S. (2023). Clinical success of guided tissue regeneration for treating vertical bone and furcation defects in dogs. Front. Vet. Sci..

[9] Christgau M., Caffesse R.G., Schmalz G., D’Souza R.N. (2007). Extracellular matrix expression and periodontal wound-healing dynamics following guided tissue regeneration therapy in canine furcation defects. J. Clin. Periodontol..

[10] Oh T.J., Meraw S.J., Lee E.J., Giannobile W.V., Wang H.L. (2003). Comparative analysis of collagen membranes for the treatment of implant dehiscence defects. Clin. Oral Implant. Res..

[11] Abe G.L., Sasaki J.I., Tsuboi R., Kohno T., Kitagawa H., Imazato S. (2024). Poly(lactic acid/caprolactone) bilayer membrane achieves bone regeneration through a prolonged barrier function. J. Biomed. Mater. Res. B Appl. Biomater..

[12] Haider T.P., Völker C., Kramm J., Landfester K., Wurm F.R. (2019). Plastics of the Future? The Impact of Biodegradable Polymers on the Environment and on Society. Angew. Chem. Int. Ed..

[13] Kaushal S., Kumar A., Khan M.A., Lal N. (2016). Comparative study of nonabsorbable and absorbable barrier membranes in periodontal osseous defects by guided tissue regeneration. J. Oral Biol. Craniofacial Res..

[14] Elharar F., Rodriguez H.J., Benqué E.P., Caffesse R.G. (1998). Guided tissue regeneration with bioabsorbable and expanded polytetrafluoroethylene barrier membranes in the treatment of naturally occurring periodontal defects in dogs. J. Periodontol..

[15] Al Salamah L., Babay N., Anil S., Al Rasheed A., Bukhary M. (2012). Guided bone regeneration using resorbable and non-resorbable membranes: A histological study in dogs. Odontostomatol. Trop..

[16] Robert P.M., Frank R.M. (1994). Periodontal guided tissue regeneration with a new resorbable polylactic acid membrane. J. Periodontol..

[17] Jin X., Park J.Y., Lee J.S., Jung U.W., Choi S.H., Cha J.K. (2023). Tissue integration patterns of non-crosslinked and crosslinked collagen membranes: An experimental in vivo study. J. Periodontal Implant Sci..

[18] Behfarnia P., Khorasani M.M., Birang R., Abbas F.M. (2012). Histological and histomorphometric analysis of animal experimental dehiscence defect treated with three bio absorbable GTR collagen membrane. Dent. Res. J..

[19] Zubery Y., Goldlust A., Alves A., Nir E. (2007). Ossification of a novel cross-linked porcine collagen barrier in guided bone regeneration in dogs. J. Periodontol..

[20] Usha R., Sreeram K.J., Mandal A.B. (2013). Organization of collagen in the presence of diphenyl phosphoryl azide (DPPA): An in vitro study. Colloids Surf. B Biointerfaces.

[21] Owens K.W., Yukna R.A. (2001). Collagen membrane resorption in dogs: A comparative study. Implant Dent..

[22] Sanz-Martin I., Ferrantino L., Vignoletti F., Nuñez J., Baldini N., Duvina M., Alcaraz J., Sanz M. (2018). Contour changes after guided bone regeneration of large non-contained mandibular buccal bone defects using deproteinized bovine bone mineral and a porcine-derived collagen membrane: An experimental in vivo investigation. Clin. Oral Investig..

[23] Kantarci A., Hasturk H., Van Dyke T.E. (2015). Animal models for periodontal regeneration and peri-implant responses. Periodontol. 2000.

[24] Lee S.H., An S.J., Lim Y.M., Huh J.B. (2017). The Efficacy of Electron Beam Irradiated Bacterial Cellulose Membranes as Compared with Collagen Membranes on Guided Bone Regeneration in Peri-Implant Bone Defects. Materials.

[25] Li X., Wang X., Zhao T., Gao B., Miao Y., Zhang D., Dong Y. (2014). Guided Bone Regeneration Using Chitosan-Collagen Membranes in Dog Dehiscence-Type Defect Model. J. Oral Maxillofac. Surg..

[26] Lee C.H., Singla A., Lee Y. (2001). Biomedical applications of collagen. Int. J. Pharm..

[27] Crigger M., Bogle G.C., Garrett S., Gantes B.G. (1996). Repair Following Treatment of Circumferential Periodontal Defects in Dogs with Collagen and Expanded Polytetrafluoroethylene Barrier Membranes. J. Periodontol..

[28] Pla R., Sanz-Esporrin J., Noguerol F., Vignoletti F., Gamarra P., Sanz M. (2023). A Synthetic Bio-Absorbable Membrane in Guided Bone Regeneration in Dehiscence-Type Defects: An Experimental In Vivo Investigation in Dogs. Bioengineering.

[29] Xie X., Shi X., Wang S., Cao L., Yang C., Ma Z. (2020). Effect of Attapulgite-Doped Electrospun Fibrous PLGA Scaffold on Pro-Osteogenesis and Barrier Function in the Application of Guided Bone Regeneration. Int. J. Nanomed..

[30] Iglhaut G., Schwarz F., Gründel M., Mihatovic I., Becker J., Schliephake H. (2014). Shell technique using a rigid resorbable barrier system for localized alveolar ridge augmentation. Clin. Oral Implant. Res..

[31] Ge J., Yang C., Wang Y., Zheng J., Hua H., Zhu J. (2018). Comparison of different grafting materials for treatment of bone defect distal to the molar in canine. Clin. Implant Dent. Relat. Res..

[32] Rothamel D., Schwarz F., Fienitz T., Smeets R., Dreiseidler T., Ritter L., Happe A., Zöller J. (2012). Biocompatibility and biodegradation of a native porcine pericardium membrane: Results of in vitro and in vivo examinations. Int. J. Oral Maxillofac. Implant..

[33] Schwarz F., Ferrari D., Podolsky L., Mihatovic I., Becker J. (2010). Initial pattern of angiogenesis and bone formation following lateral ridge augmentation using rhPDGF and guided bone regeneration: An immunohistochemical study in dogs. Clin. Oral Implant. Res..

[34] Vahdatinia F., Hooshyarfard A., Jamshidi S., Shojaei S., Patel K., Moeinifard E., Haddadi R., Farhadian M., Gholami L., Tayebi L. (2023). 3D-Printed Soft Membrane for Periodontal Guided Tissue Regeneration. Materials.

[35] Cha J.K., Joo M.J., Yoon S., Lee J.S., Choi S.H., Jung U.W. (2017). Sequential healing of onlay bone grafts using combining biomaterials with cross-linked collagen in dogs. Clin. Oral Implant. Res..

[36] Bornstein M.M., Bosshardt D., Buser D. (2007). Effect of two different bioabsorbable collagen membranes on guided bone regeneration: A comparative histomorphometric study in the dog mandible. J. Periodontol..

[37] Won J.Y., Park C.Y., Bae J.H., Ahn G., Kim C., Lim D.H., Cho D.W., Yun W.S., Shim J.H., Huh J.B. (2016). Evaluation of 3D printed PCL/PLGA/β-TCP versus collagen membranes for guided bone regeneration in a beagle implant model. Biomed. Mater..

[38] Seo G.Y., Thoma D.S., Jung U.W., Lee J.S. (2019). Increasing the tissue thickness at implant sites using guided bone regeneration and an additional collagen matrix: Histologic observations in beagle dogs. J. Biomed. Mater. Res. B Appl. Biomater..

[39] Paik J.W., Kwon Y.H., Park J.Y., Jung R.E., Jung U.W., Thoma D.S. (2024). Effect of Membrane Fixation and the Graft Combinations on Horizontal Bone Regeneration: Radiographic and Histologic Outcomes in a Canine Model. Biomater. Res..

[40] Lee B.-S., Lee C.-C., Lin H.-P., Shih W.-A., Hsieh W.-L., Lai C.-H., Takeuchi Y., Chen Y.-W. (2016). A functional chitosan membrane with grafted epigallocatechin-3-gallate and lovastatin enhances periodontal tissue regeneration in dogs. Carbohydr. Polym..

[41] Dai P., Qi G., Zhu M., Du Q., Wang K., Gao Y., Li M., Feng X., Zhang X. (2024). Periodontal ligament stem cell tissue engineering scaffolds can guide and promote canine periodontal tissue regeneration. Front. Vet. Sci..

[42] Rajwade J.M., Paknikar K.M., Kumbhar J.V. (2015). Applications of bacterial cellulose and its composites in biomedicine. Appl. Microbiol. Biotechnol..

[43] Zare Y., Shabani I. (2016). Polymer/metal nanocomposites for biomedical applications. Mater. Sci. Eng. C.

[44] Li G., Zhao M., Xu F., Yang B., Li X., Meng X., Teng L., Sun F., Li Y. (2020). Synthesis and Biological Application of Polylactic Acid. Molecules.

[45] Bogle G., Garrett S., Stoller N.H., Swanbom D.D., Fulfs J.C., Rodgers P.W., Whitman S., Dunn R.L., Southard G.L., Polson A.M. (1997). Periodontal regeneration in naturally occurring Class II furcation defects in beagle dogs after guided tissue regeneration with bioabsorbable barriers. J. Periodontol..

[46] Coonts B.A., Whitman S.L., O’Donnell M., Polson A.M., Bogle G., Garrett S., Swanbom D.D., Fulfs J.C., Rodgers P.W., Southard G.L. (1998). Biodegradation and biocompatibility of a guided tissue regeneration barrier membrane formed from a liquid polymer material. J. Biomed. Mater. Res..

[47] Araújo M.G., Berglundh T., Lindhe J. (1998). GTR treatment of degree III furcation defects with 2 different resorbable barriers. An experimental study in dogs. J. Clin. Periodontol..

[48] Suaid F.F., Ribeiro F.V., Rodrigues T.L., Silvério K.G., Carvalho M.D., Nociti F.H., Casati M.Z., Sallum E.A. (2011). Autologous periodontal ligament cells in the treatment of class II furcation defects: A study in dogs. J. Clin. Periodontol..

[49] Lindhe J., Pontoriero R., Berglundh T., Araujo M. (1995). The effect of flap management and bioresorbable occlusive devices in GTR treatment of degree III furcation defects. J. Clin. Periodontol..

[50] Suaid F.F., Ribeiro F.V., Gomes T.R., Silvério K.G., Carvalho M.D., Nociti F.H., Casati M.Z., Sallum E.A. (2012). Autologous periodontal ligament cells in the treatment of Class III furcation defects: A study in dogs. J. Clin. Periodontol..

[51] Hua N., Ti V.L., Xu Y. (2014). Biodegradable effect of PLGA membrane in alveolar bone regeneration on beagle dog. Cell Biochem. Biophys..

[52] Chang C.Y., Yamada S. (2000). Evaluation of the regenerative effect of a 25% doxycycline-loaded biodegradable membrane for guided tissue regeneration. J. Periodontol..

[53] Matsumoto G., Hoshino J., Kinoshita Y., Sugita Y., Kubo K., Maeda H., Ikada Y., Kinoshita Y. (2012). Alveolar bone regeneration using poly-(lactic acid-co-glycolic acid-co-ε-caprolactone) porous membrane with collagen sponge containing basic fibroblast growth factor: An experimental study in the dog. J. Biomater. Appl..

[54] Jung R.E., Lecloux G., Rompen E., Ramel C.F., Buser D., Hammerle C.H. (2009). A feasibility study evaluating an in situ formed synthetic biodegradable membrane for guided bone regeneration in dogs. Clin. Oral Implant. Res..

[55] Abe G.L., Tsuboi R., Kitagawa H., Sasaki J.I., Li A., Kohno T., Imazato S. (2022). Poly(lactic acid/caprolactone) bilayer membrane blocks bacterial penetration. J. Periodontal Res..

[56] Gotfredsen K., Nimb L., Hjørting-hansen E. (1994). Immediate implant placement using a biodegradable barrier, polyhydroxybutyrate-hydroxyvalerate reinforced with polyglactin 910. An experimental study in dogs. Clin. Oral Implant. Res..

[57] von Arx T., Cochran D.L., Schenk R.K., Buser D. (2002). Evaluation of a prototype trilayer membrane (PTLM) for lateral ridge augmentation: An experimental study in the canine mandible. Int. J. Oral Maxillofac. Surg..

[58] Wang D., Zhou X., Cao H., Zhang H., Wang D., Guo J., Wang J. (2023). Barrier membranes for periodontal guided bone regeneration: A potential therapeutic strategy. Front. Mater..

[59] Kikuchi M., Koyama Y., Takakuda K., Miyairi H., Shirahama N., Tanaka J. (2002). In vitro change in mechanical strength of beta-tricalcium phosphate/copolymerized poly-L-lactide composites and their application for guided bone regeneration. J. Biomed. Mater. Res..

[60] Kurtiş B., Unsal B., Cetiner D., Gültekin E., Ozcan G., Celebi N., Ocak O. (2002). Effect of polylactide/glycolide (PLGA) membranes loaded with metronidazole on periodontal regeneration following guided tissue regeneration in dogs. J. Periodontol..

[61] Reis E.C., Borges A.P., del Carlo R.J., Oliveira P.M., Sepúlveda R.V., Fernandes N.A., Martins L.M., Carvalho T.B. (2013). Guided tissue regeneration using rigid absorbable membranes in the dog model of chronic furcation defect. Acta Odontol. Scand..

[62] Kikuchi M., Koyama Y., Yamada T., Imamura Y., Okada T., Shirahama N., Akita K., Takakuda K., Tanaka J. (2004). Development of guided bone regeneration membrane composed of β-tricalcium phosphate and poly (l-lactide-co-glycolide-co-ε-caprolactone) composites. Biomaterials.

[63] Zhou T., Liu X., Sui B., Liu C., Mo X., Sun J. (2017). Development of fish collagen/bioactive glass/chitosan composite nanofibers as a GTR/GBR membrane for inducing periodontal tissue regeneration. Biomed. Mater..

[64] He H., Yu J., Cao J., E L., Wang D., Zhang H., Liu H. (2011). Biocompatibility and Osteogenic Capacity of Periodontal Ligament Stem Cells on nHAC/PLA and HA/TCP Scaffolds. J. Biomater. Sci. Polym. Ed..

[65] Jung R.E., Kokovic V., Jurisic M., Yaman D., Subramani K., Weber F.E. (2011). Guided bone regeneration with a synthetic biodegradable membrane: A comparative study in dogs. Clin. Oral Implant. Res..

[66] Kwon D.H., Bennett W., Herberg S., Bastone P., Pippig S., Rodriguez N.A., Susin C., Wikesjö U.M. (2010). Evaluation of an injectable rhGDF-5/PLGA construct for minimally invasive periodontal regenerative procedures: A histological study in the dog. J. Clin. Periodontol..

[67] Raja I.S., Preeth D.R., Vedhanayagam M., Hyon S.-H., Lim D., Kim B., Rajalakshmi S., Han D.-W. (2021). Polyphenols-loaded electrospun nanofibers in bone tissue engineering and regeneration. Biomater. Res..

[68] Dawson J.I., Oreffo R.O. (2013). Clay: New opportunities for tissue regeneration and biomaterial design. Adv. Mater..

[69] Gawor J.P., Strøm P., Nemec A. (2022). Treatment of Naturally Occurring Periodontitis in Dogs with a New Bio-Absorbable Regenerative Matrix. Front. Vet. Sci..

[70] Lekovic V., Klokkevold P.R., Kenney E.B., Dimitrijelic B., Nedic M., Weinlaender M. (1998). Histologic evaluation of guided tissue regeneration using 4 barrier membranes: A comparative furcation study in dogs. J. Periodontol..

[71] Rice C.A., Snyder C.J., Soukup J.W. (2012). Use of an autogenous cortical graft in combination with guided tissue regeneration for treatment of an infrabony defect. J. Vet. Dent..

[72] Bebel A., Rochette J. (2019). Guided Tissue Regeneration Therapy with Bone Augmentation in a Lingual, Infrabony Osseous Defect of a Mandibular Canine. J. Vet. Dent..

[73] Niemiec B.A. (2008). Periodontal Disease. Top. Companion Anim. Med..

[74] Gingerich W., Stepaniuk K. (2011). Guided tissue regeneration for infrabony pocket treatment in dogs. J. Vet. Dent..

[75] Stepaniuk K.S., Gingerich W. (2015). Evaluation of an Osseous Allograft Membrane for Guided Tissue Regeneration in the Dog. J. Vet. Dent..

[76] Alterman J.B., Huff J.F. (2016). Guided Tissue Regeneration in Four Teeth Using a Liquid Polymer Membrane. J. Vet. Dent..

[77] Barthel R.E. (2006). Treatment of vertical bone loss in a dog. J. Vet. Dent..

[78] Baumgartner D., Schramel J.P., Kau S., Unger E., Oberoi G., Peham C., Eberspächer-Schweda M. (2023). 3D printed plates based on generative design biomechanically outperform manual digital fitting and conventional systems printed in photopolymers in bridging mandibular bone defects of critical size in dogs. Front. Vet. Sci..

[79] Shim J.H., Won J.Y., Park J.H., Bae J.H., Ahn G., Kim C.H., Lim D.H., Cho D.W., Yun W.S., Bae E.B. (2017). Effects of 3D-Printed Polycaprolactone/β-Tricalcium Phosphate Membranes on Guided Bone Regeneration. Int. J. Mol. Sci..

